# ANGPTL2+cancer-associated fibroblasts and SPP1+macrophages are metastasis accelerators of colorectal cancer

**DOI:** 10.3389/fimmu.2023.1185208

**Published:** 2023-08-24

**Authors:** Xiangxiang Liu, Jian Qin, Junjie Nie, Rui Gao, Shangshang Hu, Huiling Sun, Shukui Wang, Yuqin Pan

**Affiliations:** ^1^ General Clinical Research Center, Nanjing First Hospital, Nanjing Medical University, Nanjing, Jiangsu, China; ^2^ Division of Clinical Pharmacy, General Clinical Research Center, Nanjing First Hospital, China Pharmaceutical University, Nanjing, Jiangsu, China; ^3^ Jiangsu Collaborative Innovation Center on Cancer Personalized Medicine, Nanjing Medical University, Nanjing, Jiangsu, China

**Keywords:** colorectal cancer, liver metastasis, single-cell RNA sequencing, cancer-associated fibroblast, macrophage

## Abstract

**Background:**

Liver metastasis (LM) is a leading cause of cancer-related deaths in CRC patients, whereas the associated mechanisms have not yet been fully elucidated. Therefore, it is urgently needed to deeply explore novel metastasis accelerators and therapeutic targets of LM-CRC.

**Methods:**

The bulk RNA sequencing data and clinicopathological information of CRC patients were enrolled from the TCGA and GEO databases. The single-cell RNA sequencing (scRNA-seq) datasets of CRC were collected from and analyzed in the Tumor Immune Single-cell Hub (TISCH) database. The infiltration levels of cancer-associated fibroblasts (CAFs) and macrophages in CRC tissues were estimated by multiple immune deconvolution algorithms. The prognostic values of genes were identified by the Kaplan-Meier curve with a log-rank test. GSEA analysis was carried out to annotate the significantly enriched gene sets. The biological functions of cells were experimentally verified.

**Results:**

In the present study, hundreds of differentially expressed genes (DEGs) were selected in LM-CRC compared to primary CRC, and these DEGs were significantly associated with the regulation of endopeptidase activity, blood coagulation, and metabolic processes. Then, SPP1, CAV1, ANGPTL2, and COLEC11 were identified as the characteristic DEGs of LM-CRC, and higher expression levels of SPP1 and ANGPTL2 were significantly associated with worse clinical outcomes of CRC patients. In addition, ANGPTL2 and SPP1 mainly distributed in the tumor microenvironment (TME) of CRC tissues. Subsequent scRNA-seq analysis demonstrated that ANGPTL2 and SPP1 were markedly enriched in the CAFs and macrophages of CRC tissues, respectively. Moreover, we identified the significantly enriched gene sets in LM-CRC, especially those in the SPP1^+^macrophages and ANGPTL2^+^CAFs, such as the HALLMARK_EPITHELIAL_MESENCHYMAL_TRANSITION and the HALLMARK_COMPLEMENT. Finally, our *in vitro* experiments proved that ANGPTL2^+^CAFs and SPP1^+^macrophages promote the metastasis of CRC cells.

**Conclusion:**

Our study selected four characteristic genes of LM-CRC and identified ANGPTL2^+^CAFs and SPP1^+^macrophages subtypes as metastasis accelerators of CRC which provided a potential therapeutic target for LM-CRC.

## Introduction

Colorectal cancer (CRC), one of the most common malignancies, accounts for the third incidence and second mortality rates among all tumors worldwide (1). Liver metastasis (LM) is responsible for the major deaths of CRC patients (1, 2). Although the therapeutic strategies develop, the clinical outcomes of LM-CRC patients remain unfavorable (3). The 5-year survival rate of early CRC patients who undergo complete surgical resection can reach 30% - 57%, while the 5-year survival rate of LM-CRC patients is less than 5% (4). Therefore, it is urgently needed to deeply explore the metastatic drivers and therapeutic targets of LM-CRC.

Cancer cells live in a complex microenvironment named the tumor microenvironment (TME) which contains stromal cells, endothelial cells, and immune cells (5). Mounting evidence has proved that TME is the stimulator of the progression of cancer (6). Monocyte-derived macrophages (M0) are one of the well-characterized innate immune cells (7). Sometimes, it can make up to 50 percent of the tumor mass (5). Typically, M0 macrophages can be polarized into classically activated M1 and alternatively activated M2 subtypes which are collectively termed tumor-associated macrophages (TAMs) (8). TAMs promote tumor cell proliferation, invasion, and metastasis (9). For example, TAMs can stimulate angiogenesis and suppress the antitumor function of immune T cells (10). Cancer-associated fibroblasts (CAFs) are another important component of TME and secrete growth factors, inflammatory ligands, and extracellular matrix proteins (11). CAFs are significantly associated with the metastasis of cancers. For instance, CAF-derived IL-32 can promote the invasion and metastasis of breast cancer cells via p38/MAPK signaling (12). Single-cell RNA sequencing (scRNA-seq) is an optimized high-throughput sequencing technology that can define the global gene expression of a single cell and facilitate the dissection of the heterogeneity of cancer tissues (13). Li et al. determined that there are two different CAF subtypes in the TME of CRC carcinoma tissues through scRNA-seq analysis (14). In addition, Lin et al. reported that CTSB^+^ macrophages repress the memory immune hub in the liver metastasis site of CRC (15). However, LM-associated subtypes of macrophages and CAFs remain elusive in CRC and deserve further investigation.

In this study, we identified four characteristic genes of LM-CRC and found that SPP1 and ANGPTL2 were significantly associated with the prognosis of CRC patients and mainly located at the TME. Furthermore, scRNA-seq analysis showed that SPP1 and ANGPTL2 were distributed in the macrophages and CAFs in CRC tissues, respectively. In addition, we identified the significantly enriched gene sets in LM-CRC, especially those in the SPP1^+^macrophages and ANGPTL2^+^CAFs. Finally, the metastasis accelerator roles of ANGPTL2^+^CAFs and SPP1^+^macrophages were experimentally verified.

## Materials and methods

### Bulk RNA-seq data analysis

The bulk RNA sequencing data of CRC tissues were downloaded from the TCGA CRC cohort (http://tcga.xenahubs.net) and four GEO datasets (GSE81558, GSE49355, GSE178120 and GSE159216) (16–18). All expression values of genes have been normalized. The differentially expressed genes (DEGs) were identified by the R “limma” package with a criterion of |log_2_FC|≥1 and p<0.05. The clinicopathological characteristics and prognostic information of CRC patients were downloaded from the TCGA database. The GSE81558 dataset contains 23 primary CRC tissues and 19 LM-CRC tissues. The GSE49355 dataset consists of 20 primary CRC tissues and 19 LM-CRC tissues. The GSE178120 dataset contains 105 primary CRC tissues. The GSE159216 dataset consists of 283 LM-CRC tissues. The TCGA CRC cohort includes 362 CRC patients with follow-up times.

### ScRNA-seq data analysis

The scRNA-seq data of CRC tissues were enrolled from a GEO scRNA-seq dataset (GSE166555) (19) and an EMTAB scRNA-seq dataset (EMTAB8107) that were deposited in the public Tumor Immune Single-cell Hub (TISCH) database (http://tisch.comp-genomics.org/home/) (20). The values in the single-cell level expression matrix are normalized by the NormalizeData method in “Seurat” to scale the raw counts (UMI) in each cell to 10,000. A uniform analysis algorithm (MAESTRO) was adopted for each dataset to perform quality control, clustering, and cell-type annotation. The GSE166555 dataset contains 66,050 cells from 12 CRC tissues. The EMTAB8107 dataset consists of 23,176 cells from 7 CRC tissues. The scRNA-seq was conducted by the platform of 10×Genomics.

### Functional enrichment analysis

Gene Ontology (GO) and Kyoto Encyclopedia of Genes and Genomes (KEGG) pathway analyses were carried out in the online DAVID database (https://david.ncifcrf.gov). GO analysis includes the biological process (BP), molecular function (MF), and cellular component (CC) categories. The results of the GO and KEGG analyses were further visualized through an online tool, OmicShare (http://www.omicshare.com/tools). The GSEA analysis was carried out based on the MSigDB database (http://www.gsea-msigdb.org/gsea/msigdb/index.jsp). The significantly enriched gene sets were selected with a criterion of |NES|>1, nominal (NOM) p-value<0.05, and false discovery rate (FDR) value<0.25. GSEA 4.3.0 software was used for enrichment analysis.

### Evaluating the infiltration levels of CAFs and macrophages

The infiltration abundances of CAFs and macrophages were estimated in the online tool TIMER2 (http://timer.cistrome.org) by multiple immune deconvolution methods. The infiltration levels of macrophages were assessed by the TIMER, CIBERSORT, CIBERSORT-ABS, EPIC, QUANTISEQ, MCPCOUNTER, and XCELL algorithms. The infiltration levels of CAFs were assessed by the MCPCOUNTER, EPIC, and XCELL algorithms.

### Collecting tissue samples

Ten paired CRC tissues and corresponding normal tissues were obtained from CRC patients undergoing tumor resection. The clinicopathological characteristics of the CRC tissues were described in **Table 1**. All enrolled patients signed the informed consent and did not receive any anti-tumor treatment previously. This study was approved by the Ethics Committee of Nanjing First Hospital.

**Table 1:** The clinicopathological characteristics of enrolled CRC tissues.

Number	Position	Age	Gender	pTNM
1	Colon	75	Male	T3N0M0
2	Colon	71	Female	T1N0M0
3	Colon	56	Male	T2N1aM0
4	Rectum	68	Female	T1N0M0
5	Colon	71	Male	T3N1aM0
6	Rectum	58	Male	T2N0M0
7	Colon	57	Male	T3N0M0
8	Colon	70	Female	T3N2aM0
9	Colon	62	Male	T3N2aM0
10	Colon	59	Male	T3N0M0

### Isolating fibroblasts

Fibroblasts were isolated from fresh tissues as previously described (21). Briefly, the minced tissue samples were digested in the mixture of 1mg/mL collagenase (cat. #C4-BIOC, Sigma-Aldrich), Dulbecco’s modified Eagle’s medium (DMEM; cat. #KGM12800, KeyGen), and 10% fetal bovine serum (FBS; cat. #12106C, Sigma, Sigma-Aldrich) for 2 h at 37 °C with shaking. After centrifugation, cell pellets were resuspended and further filtered through a cell strainer (100 μm). Then cells were cultured in DMEM containing 10% FBS. Two hours later, change culture medium. Compared to other cells, fibroblasts are easier to attach to the culture dishes. Fibroblasts were identified by the detection of two positive markers (α-SMA and Vimentin) and two negative markers (KRT20 and Desmin) (22).

### Cell culture

HCT116, HCT8, and THP1 cells were purchased from the American Types Culture Collection (ATCC) and identified by short tandem repeat (STR) analysis. CRC cells, CAFs, and NFs were cultured in DMEM complete medium (cat. #KGM12800, KeyGen) supplemented with 10% FBS, 100U/mL penicillin, and 0.1 mg/mL streptomycin. THP1 cells were cultured in RPMI-1640 medium supplemented with 10% FBS, 1% penicillin, and 1% streptomycin. The THP-1 monocytes were differentiated into macrophages with 10 ng/ml phorbol-12-myristate-13-acetate (PMA) for 48 h (23). All cells grew in a humidified atmosphere containing 5% CO2 at 37 °C.

### ELISA assay

The protein levels of ANGPTL2 in CAFs and NFs culture media were measured by an ELISA kit (cat. #CSB-E13881h, CUSABIO). Briefly, an equal volume of cell culture medium from the same number of CAFs or NFs was centrifugated for 15 min at 1000 × g and 4°C, and the supernate was collected and kept on ice. Add 100 μL of standard and sample per well and cover with the adhesive strip and incubate for 2 h at 37°C. Then remove the liquid from each well without washing. Add 100 μL of Biotin-antibody (1×) to each well, cover with a new adhesive strip, and incubate for 1 h at 37°C. Aspirate each well and wash for a total of three washes. Next, wash each well with 200 μL Wash Buffer and let it stand for 2 min. After the last wash, remove any remaining Wash Buffer and add 100 μL of HRP-avidin (1×) to each well. Cover the microtiter plate with a new adhesive strip and incubate for 1 h at 37°C. Subsequently, add 90 μL of TMB substrate to each well and incubate for 30 min at 37°C in the dark. Add 50 μL of Stop Solution to each well, and gently tap the plate to ensure thorough mixing. Finally, the absorbance (450nm) of each well was detected by a microplate reader (Infinite M200 PRO, TECAN).

### Cell transfection

The SPP1 cDNAs were cloned into the multi-cloning site of Lentiviral pLVX plasmid (SyngenTech, Beijing, China) and verified by sequencing. Then, pLVX-SPP1 plasmids were transfected into macrophages using Lipofectamine 3000 (cat. # L3000001, Invitrogen). SPP1 overexpressing macrophages were selected after treatment with 10 μg/mL puromycin (cat. # ST551, Beyotime) for seven days. The recombinant human ANGPTL2 protein (rANGPTL2, cat. # H00023452-P01, Abnova) was added in the culture medium of CAFs to overexpress ANGPTL2.

### Immunofluorescence (IF) assay

The CAFs and macrophages seeded on sterile coverslips in 24-well plates were washed thrice with PBS and fixed with 4% paraformaldehyde for 15 min. Then cells were permeabilized with 0.1% Triton X-100 for 15 min and incubated with 2% BSA for 1 h. Next, cells were incubated with primary antibodies anti-ANGPTL2 (1:200, cat. # 12316-1-AP, Proteintech), anti-α-SMA (1:100, cat. # ab240654, Abcam), anti-CD68 (1:200, cat. # 28058-1-AP, Proteintech), anti-SPP1 (1:200, cat. # sc-21742, Santa Cruz), anti-vimentin (1:200, cat. #ab92547, Abcam), anti-KRT20 (1:100, cat. # ab76126, Abcam), or anti-Desmin (1:200, cat. # ab32362, Abcam) at 4°C overnight. After washing thrice, the corresponding secondary antibody goat anti-rabbit IgG H&L (Alexa Fluor^®^ 647) (1:400, cat. #ab150079, Abcam) or goat anti-mouse IgG H&L (Alexa Fluor^®^ 488) (1:400, cat. #ab150113, Abcam) was added, and the cells were incubated at room temperature for 1 h. Nuclei were counterstained with DAPI (cat. #P0131, Beyotime). Images were obtained using a microscope (Imager. A2, ZEISS).

### Western blot assay

The total proteins of cells were extracted by RIPA lysis buffer (cat. # P0013C, Beyotime), and protein concentrations were quantified using a bicinchoninic acid (BCA) kit (cat. # KGP902, KeyGEN). Then proteins were separated by sodium dodecyl sulfate-polyacrylamide gel electrophoresis (SDS-PAGE) and transferred onto polyvinylidene difluoride (PVDF) membranes (cat. #IPVH00010, Millipore). Subsequently, the PVDF membranes were blocked with 5% skim milk and incubated with the corresponding primary antibody at 4°C overnight. After washing thrice with tris-buffered saline with tween-20 (TBST), the membranes were incubated with horseradish peroxidase (HRP)-conjugated secondary antibodies at room temperature for 2 h. Blots were visualized using BeyoECL Plus (cat. #P0018S, Beyotime). The antibodies used in this study were as follows: anti-ANGPTL2 (1:1000, cat. #12316-1-AP, Proteintech), anti-E-cadherin (1:10000, cat. #ab40772, Abcam), anti-N-cadherin (1:5000, cat. #ab76011, Abcam), anti-vimentin (1:2000, cat. #ab92547, Abcam), anti-SPP1 (1:1000, cat. # sc-21742, Santa Cruz), and anti-GAPDH (1:20000, cat. #60004-1-Ig, Proteintech).

### Transwell assay

For the Transwell assay, NFs, CAFs or macrophages were seeded on the lower well and cultured in 500μL DMEM or RPMI-1640 medium supplemented with 10% FBS. CRC cells (2×10^4^) were resuspended in 200μL DMEM and seeded into the upper well of 8-μm-pore chambers (cat. #3422, Corning) which had been coated with Matrigel (cat. #356234, Corning). After co-culture at 37°C and 5% CO2 for 24h, CRC cells at the upper surface of the filter were removed and the cells that invaded the opposite side of the filter were stained with 0.5% crystal violet and counted under a microscope (Nikon, Japan).

### Immunohistochemistry (IHC) assay

The IHC assay was performed on paraffin sections (5 μm) of normal tissues and CRC tissues. Briefly, after being deparaffinized and rehydration, the antigen of the sections was retrieved using 0.01 M sodium citrate buffer (pH 6.0) at a boiling temperature for 10 min. Thereafter, the sections were incubated with 3% hydrogen peroxide for 10 min and 5% bovine serum albumin for 1 h. Then, the sections were incubated with primary antibodies at 4 °C overnight. The next day, the sections were washed thrice with PBS and incubated with corresponding secondary antibodies. Finally, the DAB system was applied to visualize the signal, and hematoxylin was applied to stain the nucleus. The immunostaining images were captured using a microscope (Nikon, Japan). The degree of positivity was initially classified according to scoring both the proportion of positive staining tumor cells and the staining intensities. The IHC score was calculated as previously described (24). The antibodies used in this study were as follows: anti-CAV1 (1:200, cat. # 16447-1-AP, Proteintech), anti-SPP1 (1:200, cat. # sc-21742, Santa Cruz), anti-ANGPTL2 (1:200, cat. # 12316-1-AP, Proteintech), anti-COLEC11 (1:200, cat. # 15269-1-AP, Proteintech).

### Statistical analysis

Statistical analysis was performed by using GraphPad Prism 8.0 (GraphPad, United States) and R 4.2.0 software. The Kaplan-Meier (KM) curve with a log-rank test was used to compare the different overall survival and disease-free survival of patients between the two groups. The statistical difference between the two groups was analyzed through the Wilcoxon test. The correlations between genes and cell infiltration levels were calculated by Pearson correlation analysis. P-value<0.05 was considered statistically significant.

## Results

### Functional enrichment analysis of The DEGs between LM-CRC tissues and primary CRC tissues

**Figure 1:**
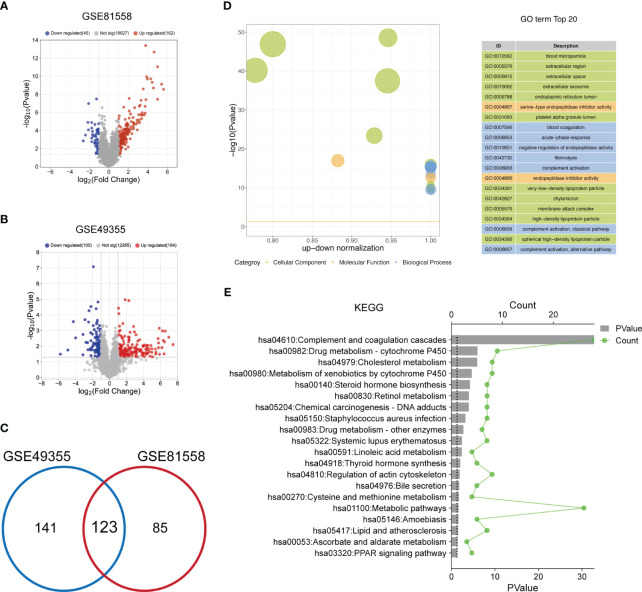
The functional enrichment analysis of DEGs between LM-CRC tissues and primary CRC tissues. **(A)** The volcano plot of the DEGs in LM-CRC based on the GSE81558 dataset. **(B)** The volcano plot of the DEGs in LM-CRC based on the GSE49355 dataset. **(C)** Overlap the DEGs in two datasets to select the common DEGs. **(D)** The top 20 GO analysis terms of common DEGs in LM-CRC. **(E)** The top 20 KEGG pathway analysis terms of common DEGs in LM-CRC.

To select the DEGs between LM-CRC tissues and primary CRC tissues, GSE81558 and GSE49355 datasets were collected. The analysis results of the GSE81558 dataset exhibited that 162 and 46 genes are significantly upregulated and downregulated in the LM-CRC tissues, respectively (**Figure 1A**). The analysis results of the GSE49355 dataset showed that there are 164 upregulated genes and 100 downregulated genes in the LM-CRC tissues (**Figure 1B**). Then we overlapped these DEGs in two datasets and identified 123 common DEGs (**Figure 1C**). GO analysis revealed that these DEGs are significantly associated with many biological processes, such as the regulation of endopeptidase activity, blood coagulation, complement activation, and fibrinolysis (**Figure 1D**). The MF analysis showed that these DEGs mainly regulate serine-type endopeptidase inhibitor activity (**Figure 1D**). In addition, the CC analysis demonstrated that these DEGs are mainly located in the extracellular region, extracellular exosome, and extracellular space (**Figure 1D**). Moreover, the KEGG analysis revealed that these DEGs mainly participate in the complement and coagulation cascades and metabolic pathways (**Figure 1E**).

### Identify SPP1, CAV1, ANGPTL2, and COLEC11 as the characteristic DEGs of LM-CRC

**Figure 2:**
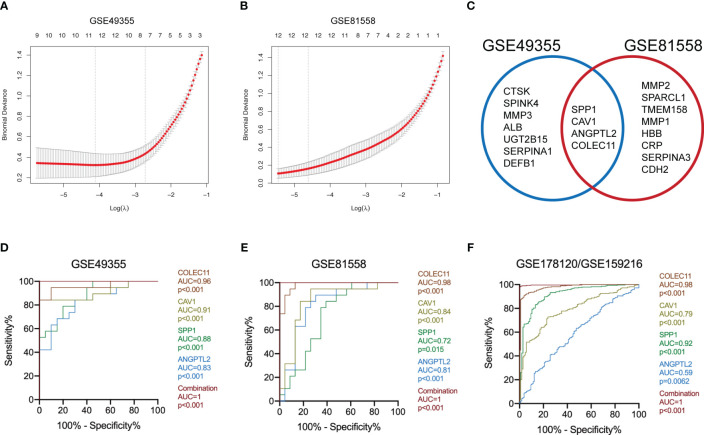
Identify the characteristic DEGs of LM-CRC. **(A)** Identify the characteristic DEGs of LM-CRC through the “LASSO” algorithm based on the GSE49355 dataset. **(B)** Identify the characteristic DEGs of LM-CRC through the “LASSO” algorithm based on the GSE81558 dataset. **(C)** Overlap the characteristic DEGs in two datasets to select the common DEGs of LM-CRC. **(D)** The ROC curves of SPP1, CAV1, ANGPTL2, COLEC11, and their combination to differentiate LM-CRC from primary CRC based on the GSE49355 dataset. **(E)** The ROC curves of SPP1, CAV1, ANGPTL2, COLEC11, and their combination to differentiate LM-CRC from primary CRC based on the GSE81558 dataset. **(F)** The ROC curves of SPP1, CAV1, ANGPTL2, COLEC11, and their combination to differentiate LM-CRC from primary CRC based on the GSE178120 and GSE159216 datasets.

The logistic “LASSO” algorithm is a shrinkage method that can actively select more relevant and interpretable predictors from a large and potentially multicollinear set of variables in the regression (25). We then selected the characteristic genes of LM-CRC from 123 DEGs through the “LASSO” algorithm. The GSE49355 dataset analysis identified 11 characteristic genes of LM (**Figure 2A**). Meanwhile, analysis of the GSE81558 dataset uncovered 12 characteristic genes of LM (**Figure 2B**). Next, we overlapped these DEGs in these datasets and selected 4 common characteristic DEGs of LM-CRC, including SPP1, CAV1, ANGPTL2, and COLEC11 (**Figure 2C**). ROC analysis of the GSE49355 dataset showed that the AUC values are 0.96, 0.91, 0.88, 0.83 for COLEC11 (p<0.001), CAV1 (p<0.001), SPP1 (p<0.001), and ANGPTL2 (p<0.001) to differentiate LM-CRC from primary CRC, respectively (**Figure 2D**). Moreover, the combination of these four genes significantly improves the AUC value to distinguish LM-CRC from primary CRC (AUC=1, p<0.001) (**Figure 2D**). ROC analysis of the GSE81558 dataset showed that the AUC values are 0.98, 0.84, 0.72, 0.81 for COLEC11 (p<0.001), CAV1 (p<0.001), SPP1 (p=0.015), and ANGPTL2 (p<0.001) to differentiate LM-CRC from primary CRC, respectively (**Figure 2E**). In addition, the AUC value of the combination of these four genes to distinguish LM-CRC from primary CRC is higher (AUC=1, p<0.001) (**Figure 2E**). The performance of the four genes in distinguishing primary and liver-metastatic CRC are further validated in independent cohorts (GSE178120 and GSE159216) which contains 105 primary CRC and 283 LM-CRC. ROC analysis showed that the AUC values are 0.98, 0.79, 0.92, 0.59 for COLEC11 (p<0.001), CAV1 (p<0.001), SPP1 (p<0.001), and ANGPTL2 (p=0.0062) to differentiate LM-CRC from primary CRC, respectively (**Figure 2F**). The AUC value of the combination of these four genes to distinguish LM-CRC from primary CRC is also higher (AUC=1, p<0.001) (**Figure 2F**).

### The protein levels and prognostic values of the characteristic DEGs of LM-CRC

**Figure 3:**
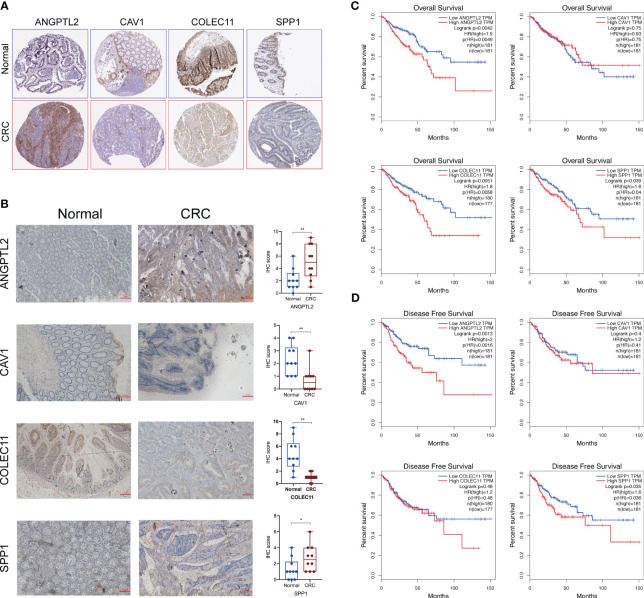
The protein levels and prognostic values of the characteristic DEGs of LM-CRC. **(A)** The protein levels of SPP1, CAV1, ANGPTL2, and COLEC11 in CRC tissues and normal tissues deposited in the Human Protein Atlas database. **(B)** The protein levels of SPP1, CAV1, ANGPTL2, and COLEC11 in CRC tissues and normal tissues revealed by IHC assay. **(C)**The association between SPP1, CAV1, ANGPTL2, and COLEC11 expression and the overall survival of CRC patients. **(D)** The association between SPP1, CAV1, ANGPTL2, and COLEC11 expression and the disease-free survival of CRC patients. *, p<0.05; **, p<0.01.

Next, we tested the protein levels of these characteristic DEGs in CRC tissues based on the Human Protein Atlas database, and the results showed that ANGPTL2 and SPP1 are upregulated in CRC tissues compared to normal tissues (**Figure 3A**). However, CAV1 and COLEC11 seem to be downregulated in CRC tissues (**Figure 3A**). Subsequently, we conducted IHC to verify their expression levels in normal and CRC tissues. Consistently, our IHC results exhibited that ANGPTL2 and SPP1 are upregulated, whereas CAV1 and COLEC11 are downregulated in CRC tissues compared to normal tissues (**Figure 3B**). Intriguingly ANGPTL2 and SPP1 are enriched in the TME but not in the CRC cells (**Figures 3A, B**). Moreover, the prognostic analysis of the TCGA CRC cohort revealed that CRC patients with higher ANGPTL2, COLEC11, and SPP1 tend to undergo worse overall survival (**Figure 3C**). In addition, elevated ANGPTL2 and SPP1 in CRC tissues are significantly associated with unfavorable disease-free survival of patients (**Figure 3D**).

### Identify ANGPTL2^+^CAFs and SPP1^+^macrophages in CRC tissues

**Figure 4:**
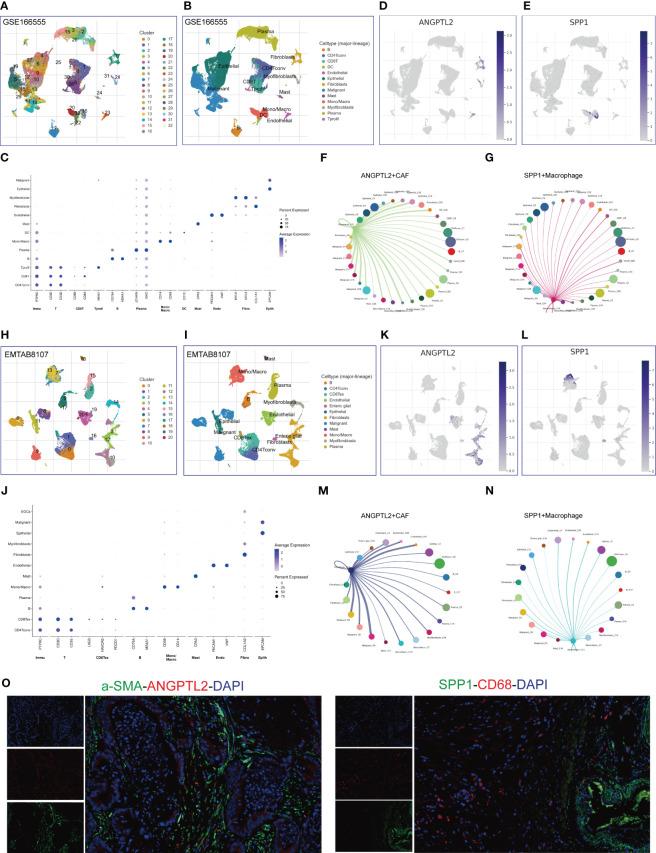
Identify ANGPTL2^+^CAFs and SPP1^+^macrophages in CRC tissues based on scRNA-seq. **(A)** The identified cell clusters in CRC tissues based on the GSE166555 dataset. **(B)** The identified cell types in CRC tissues based on the GSE166555 dataset. **(C)** The representative marker genes of identified cell types in CRC tissues based on the GSE166555 dataset. **(D)** The expression levels of ANGPTL2 in the identified cell types in CRC tissues based on the GSE166555 dataset. **(E)** The expression levels of SPP1 in the identified cell types in CRC tissues based on the GSE166555 dataset. **(F)** The interactions between ANGPTL2^+^CAFs and other cells based on the GSE166555 dataset. **(G)** The interactions between SPP1^+^macrophages and other cells based on the GSE166555 dataset. **(H)** The identified cell clusters in CRC tissues based on the EMTAB8107 dataset. **(I)** The identified cell types in CRC tissues based on the EMTAB8107 dataset. **(J)** The representative marker genes of identified cell types in CRC tissues based on the EMTAB8107 dataset. **(K)** The expression levels of ANGPTL2 in the identified cell types in CRC tissues based on the EMTAB8107 dataset. **(L)** The expression levels of SPP1 in the identified cell types in CRC tissues based on the EMTAB8107 dataset. **(M)** The interactions between ANGPTL2^+^CAFs and other cells based on the EMTAB8107 dataset. **(N)** The interactions between SPP1^+^macrophages and other cells based on the EMTAB8107 dataset. **(O)** IF assays of ANGPTL2, SPP1, fibroblast-specific marker α-SMA, and macrophage-specific marker CD68 in CRC tissues. CD8T, CD8+T cell; CD4Tconv, conventional CD4+T cell; DC, dendritic cell; Mono/Macro, Monocyte/Macrophage; Tprolif, proliferative T cell; CD8Tex, CD8+T cell exhaustion. Endo, Endothelial cell; Fibro, Fibroblast; Epith, Epithelial cell.

Given the prognostic values and unusual distribution of ANGPTL2 and SPP1 in CRC tissues, we intended to explore the cell types where they are enriched based on scRNA-seq. Analysis of the scRNA-seq data in the GSE166555 dataset showed that 33 cell clusters and 13 cell types are identified in CRC tissues (**Figures 4A, B**). The marker genes of each cell type are exhibited in **Figure 4C**. We found that ANGPTL2 is significantly enriched in the CAFs, especially in the C17 cluster (**Figure 4D**). In addition, SPP1 is significantly enriched in the mono/macrophages, especially in the C16 cluster (**Figure 4E**). The cell-cell interaction (CCI) analysis revealed that ANGPTL2^+^CAFs mainly interact with malignant CRC cells, mono/macrophages, epithelial cells, and dendritic cells (DCs) (**Figure 4F**) and that SPP1^+^macrophages mainly interact with malignant CRC cells, endothelial cells, DCs, proliferating T cells, and myofibroblasts (**Figure 4G**).

To verify our findings, we analyzed an EMTAB scRNA-seq dataset (EMTAB8107). The analysis results showed that 21 cell clusters and 12 cell types are identified in CRC tissues (**Figures 4H, I**). The marker genes of each cell type are exhibited in **Figure 4J**. Consistently, ANGPTL2 is significantly enriched in the CAFs, especially in the C10 cluster (**Figure 4K**) and SPP1 is significantly enriched in the mono/macrophages, especially in the C13 cluster (**Figure 4L**). Moreover, the CCI analysis revealed that ANGPTL2^+^CAFs mainly interact with malignant CRC cells, mono/macrophages, epithelial cells, and endothelial cells (**Figure 4M**). In addition, SPP1^+^mono/macrophages mainly interact with malignant CRC cells, endothelial cells, and exhaustive CD8+T cells (**Figure 4N**). Furthermore, we conducted IF assays to explore the distribution of ANGPTL2/SPP1 proteins and CAF/macrophage markers in CRC tissues. The results showed that ANGPTL2 and SPP1 are enriched in subcluster of CAFs/macrophages in CRC tissues, respectively (**Figure 4O**).

### Support the correlations between ANGPTL2/SPP1 and CAFs/macrophages in CRC tissues based on Bulk RNA-seq

**Figure 5:**
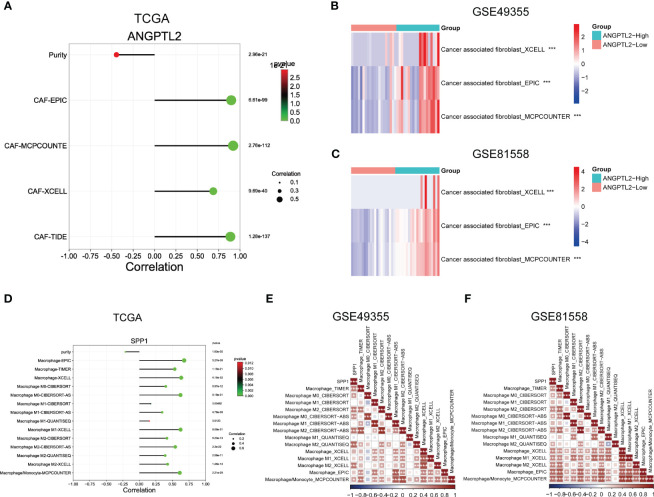
Support the correlations between ANGPTL2/SPP1 and CAFs/macrophages in CRC tissues based on bulk RNA-seq. **(A)** The correlations between ANGPTL2 expression and the purity of CRC tissue and the infiltrated level of CAFs based on the TCGA CRC cohort. **(B)** The correlations between ANGPTL2 expression and the infiltrated level of CAFs based on the GSE49355 dataset. **(C)** The correlations between ANGPTL2 expression and the infiltrated level of CAFs based on the GSE81558 dataset. **(D)** The correlations between SPP1 expression and the purity of CRC tissue and the infiltrated level of macrophages based on the TCGA CRC cohort. **(E)** The correlations between SPP1 expression and the infiltrated level of macrophages based on the GSE49355 dataset. **(F)** The correlations between SPP1 expression and the infiltrated level of macrophages based on the GSE81558 dataset. *, p<0.05; **, p<0.01; ***, p<0.001.

To support the above results, we analyzed the correlation between ANGPTL2 expression and CAFs infiltration levels as well as the correlation between SPP1 expression and macrophage infiltration levels in CRC tissues based on three bulk RNA-seq datasets. As shown in **Figure 5A**, analysis of the TCGA CRC cohort by the EPIC, MCPCOUNTE, XCELL, and TIDE algorithms showed that ANGPTL2 expression is negatively correlated with the purity of CRC tissues, but positively correlated with the infiltrated levels of CAFs. In addition, analysis of GSE49355 and GSE81558 datasets exhibited that the infiltrated levels of CAFs are significantly elevated in CRC tissues with high ANGPTL2 expression (**Figures 5B, C**). As shown in **Figure 5D**, the TCGA CRC cohort analysis by the EPIC, TIMER, MCPCOUNTE, XCELL, CIBERSORT, CIBERSORT-AS, and QUANTISEQ algorithms showed that SPP1 expression is negatively correlated with the purity of CRC tissues, but positively correlated with the infiltrated levels of multiple types of macrophages. Consistently, analysis of the GSE49355 and GSE81558 datasets showed that the infiltrated levels of macrophages, especially M2 macrophages, are positively correlated with SPP1 expression in CRC tissues (**Figures 5E, F**).

### Identify the LM-associated gene sets in ANGPTL2^+^CAFs and SPP1^+^macrophages in CRC tissues

**Figure 6:**
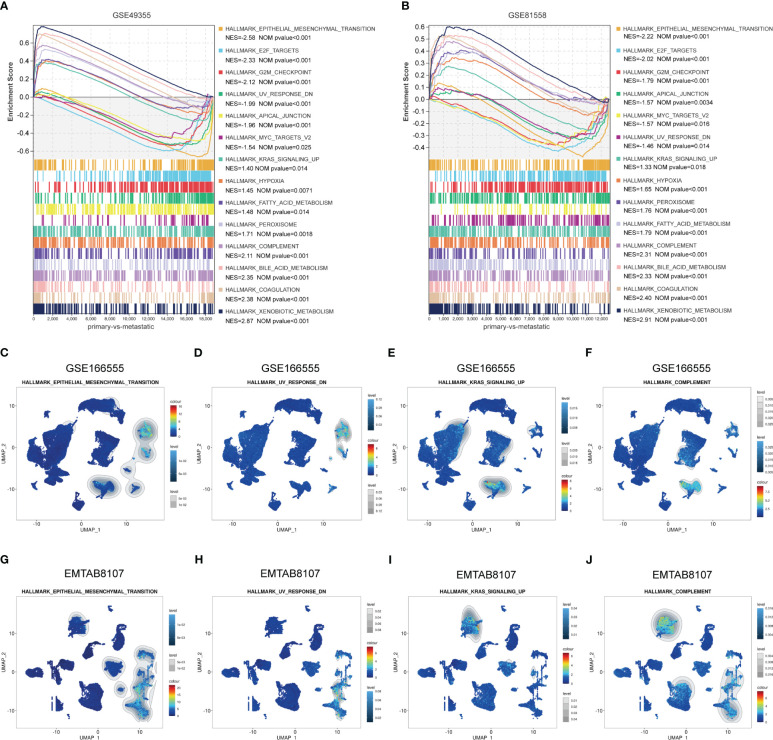
Identify the LM-associated gene sets in ANGPTL2^+^CAFs and SPP1^+^macrophages in CRC tissues. **(A)** The significantly enriched gene sets in LM-CRC based on the GSE49355 dataset. **(B)** The significantly enriched gene sets in LM-CRC based on the GSE81558 dataset. **(C)** The enrichment of the HALLMARK_EPITHELIAL_MESENCHYMAL_TRANSITION gene set in identified cell types based on the GSE166555 scRNA-seq data. **(D)** The enrichment of the HALLMARK_UV_RESPONSE_DN gene set in identified cell types based on the GSE166555 scRNA-seq data. **(E)** The enrichment of the HALLMARK_KRAS_SIGNALING_UP gene set in identified cell types based on the GSE166555 scRNA-seq data. **(F)** The enrichment of the HALLMARK_COMPLEMENT gene set in identified cell types based on the GSE166555 scRNA-seq data. **(G)** The enrichment of the HALLMARK_EPITHELIAL_MESENCHYMAL_TRANSITION gene set in identified cell types based on the EMTAB8107 scRNA-seq data. **(H)** The enrichment of the HALLMARK_UV_RESPONSE_DN gene set in identified cell types based on the EMTAB8107 scRNA-seq data. **(I)** The enrichment of the HALLMARK_KRAS_SIGNALING_UP gene set in identified cell types based on the EMTAB8107 scRNA-seq data. **(J)** The enrichment of the HALLMARK_COMPLEMENT gene set in identified cell types based on the EMTAB8107 scRNA-seq data.

To identify the enriched gene sets which are associated with LM-CRC, GSEA analysis was conducted based on the GSE49355 and GSE81558 datasets. As shown in **Figures 6A, B**, the gene sets of HALLMARK_XENOBIOTIC_METABOLISM, HALLMARK_COAGULATION, HALLMARK_BILE_ACID_ METABOLISM, HALLMARK_COMPLEMENT, HALLMARK_PEROXISOME, HALLMARK_FATTY_ACID_ METABOLISM, HALLMARK_KRAS_SIGNALING_UP, HALLMARK_HYPOXIA, HALLMARK_EPITHELIAL_MESENCHYMAL_TRANSITION, HALLMARK_E2F_TARGETS, HALLMARK_G2M_CHECKPOINT, HALLMARK_APICAL_JUNCTION, HALLMARK_MYC_TARGETS_V2, UV_RESPONSE_DN are significantly correlated with liver metastasis of CRC. Subsequently, GSEA analysis of scRNA-seq data in the GSE166555 dataset was carried out and the results uncovered that compared to other identified cell types, the gene sets of HALLMARK_EPITHELIAL_MESENCHYMAL_TRANSITION and HALLMARK_UV_RESPONSE_DN are specifically enriched in the ANGPTL2^+^CAFs of CRC tissues (**Figures 6C, D**). Besides, compared to other identified cell types, the gene sets of HALLMARK_COMPLEMENT and HALLMARK_KRAS_SIGNALING_UP are significantly enriched in the SPP1^+^macrophages of CRC tissues (**Figures 6E, F**). To validate these findings, GSEA analysis of scRNA-seq data in the EMTAB8107 dataset was conducted. Consistently, the gene sets of HALLMARK_EPITHELIAL_MESENCHYMAL_TRANSITION and HALLMARK_UV_RESPONSE_DN are specifically enriched in the ANGPTL2^+^CAFs in CRC tissues (**Figures 6G, H**) and the gene sets of HALLMARK_COMPLEMENT and HALLMARK_KRAS_SIGNALING_UP are significantly enriched in the SPP1^+^macrophages in CRC tissues (**Figures 6I, J**).

### Explore the correlations between ANGPTL2/SPP1 and ANGPTL2^+^CAFs-/SPP1^+^macrophages-specific gene sets in CRC

**Figure 7:**
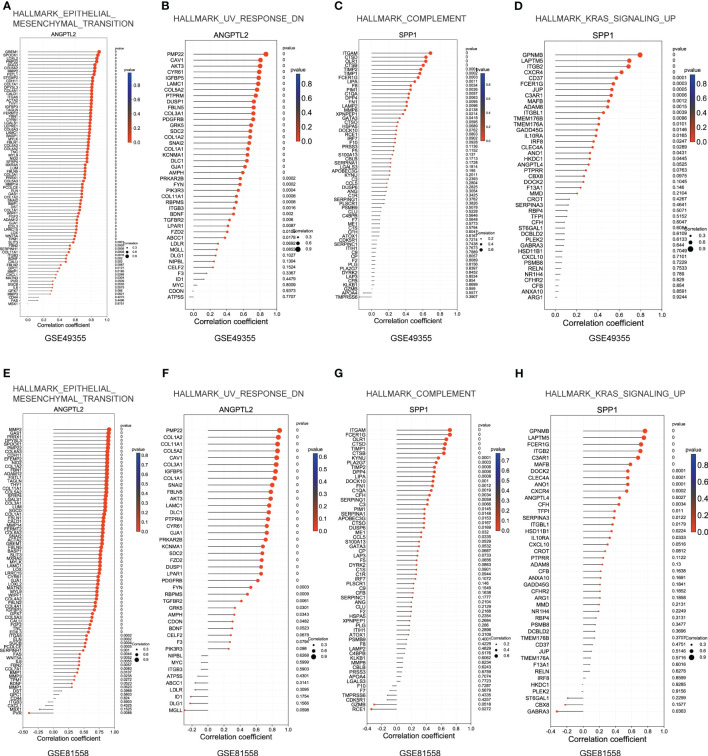
Explore the correlations between ANGPTL2/SPP1 and ANGPTL2^+^CAFs-/SPP1^+^macrophages-specific gene sets in CRC. **(A)** The correlations between ANGPTL2 expression and the HALLMARK_EPITHELIAL_MESENCHYMAL_TRANSITION gene set in CRC based on the GSE49355 dataset. **(B)** The correlations between ANGPTL2 expression and the HALLMARK_UV_RESPONSE_DN gene set in CRC based on the GSE49355 dataset. **(C)** The correlations between SPP1 expression and the HALLMARK_KRAS_SIGNALING_UP gene set in CRC based on the GSE49355 dataset. **(D)** The correlations between SPP1 expression and the HALLMARK_COMPLEMENT gene set in CRC based on the GSE49355 dataset. **(E)** The correlations between ANGPTL2 expression and the HALLMARK_EPITHELIAL_MESENCHYMAL_TRANSITION gene set in CRC based on the GSE49355 dataset. **(F)** The correlations between ANGPTL2 expression and the HALLMARK_UV_RESPONSE_DN gene set in CRC based on the GSE49355 dataset. **(G)** The correlations between SPP1 expression and the HALLMARK_KRAS_SIGNALING_UP gene set in CRC based on the GSE49355 dataset. **(H)** The correlations between SPP1 expression and the HALLMARK_COMPLEMENT gene set in CRC based on the GSE49355 dataset.

Subsequently, we explored the correlations between ANGPTL2/SPP1 and ANGPTL2^+^CAFs-/SPP1^+^macrophages-specific gene sets in CRC based on the GSE49355 and GSE81558 datasets. **Figures 7A, B** showed that the expression of most genes in the gene sets of HALLMARK_EPITHELIAL_MESENCHYMAL_TRANSITION and HALLMARK_UV_RESPONSE_DN are positively correlated with ANGPTL2 expression in CRC tissues. In addition, the expression of most genes in the gene sets of HALLMARK_COMPLEMENT and HALLMARK_KRAS_SIGNALING_UP are positively correlated with SPP1 expression in CRC tissues (**Figures 7C, D**). The GSE81558 dataset was used to validate these findings. Consistently, ANGPTL2 expression is positively correlated with the expression of most genes in the gene sets of HALLMARK_EPITHELIAL_MESENCHYMAL_TRANSITION and HALLMARK_UV_RESPONSE_DN (**Figures 7E, F**), and SPP1 expression is positively correlated with the expression of most genes in the gene sets of HALLMARK_COMPLEMENT and HALLMARK_KRAS_SIGNALING_UP (**Figures 7G, H**) in CRC tissues.

### ANGPTL2^+^CAFs promote the EMT and metastasis of CRC cells by secreting ANGPTL2

**Figure 8:**
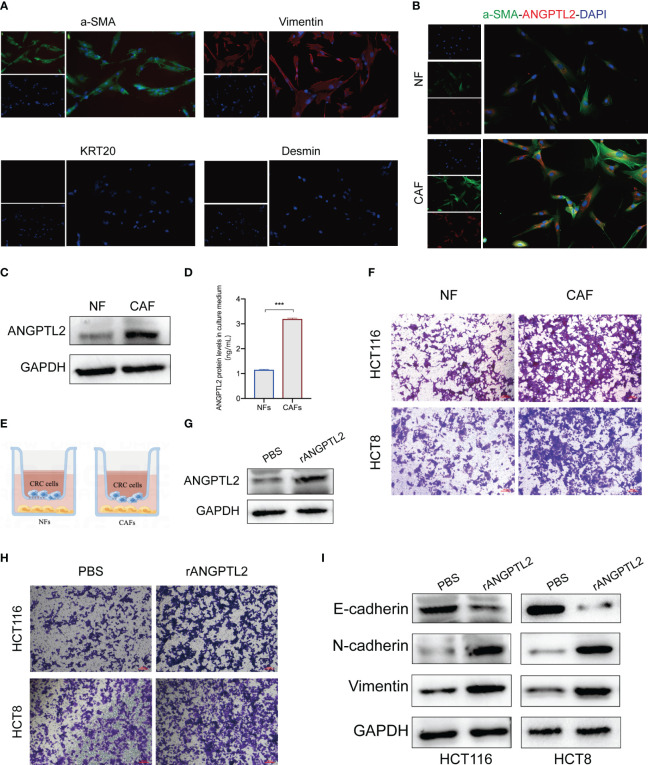
ANGPTL2^+^CAFs promote the EMT and metastasis of CRC cells by secreting ANGPTL2. **(A)** IF assays of the CAF-specific positive markers α-SMA and Vimentin and negative markers KRT20 and Desmin in fibroblasts. **(B)** IF assays of the CAF-specific markers α-SMA and ANGPTL2 in NFs and CAFs. **(C)** The protein levels of ANGPTL2 in NFs and CAFs detected by western blot assay. **(D)** The expression levels of ANGPTL2 in culture media of NFs and CAFs. **(E)** The illustration of co-culturing Fibroblasts with CRC cells. **(F)** The invasion ability of CRC cells revealed by Transwell assays. **(G)** The effect of rANGPTL2 overexpression in CAFs. **(H)** The invasion ability of CRC cells revealed by Transwell assays. **(I)** The changes of EMT-associated genes in CRC cells after co-culturing with ANGPTL2-overexpressed CAFs. ***, p<0.001.

The biological function of ANGPTL2^+^CAFs in CRC remains unclear. ANGPTL2 is a kind of secreted glycoprotein (26). Therefore, we hypothesized that ANGPTL2^+^CAFs promote the metastasis of CRC cells by secreting ANGPTL2. We first isolated fibroblasts from tissues and a panel of fibroblast markers, including two positive markers (α-SMA and Vimentin) and two negative markers (KRT20 and desmin), were applied to define the fibroblasts (**Figure 8A**). Western blot and IF assays showed that the expression of ANGPTL2 is significantly upregulated in CAFs compared to NFs (**Figures 8B, C**). ELISA assay showed that CAFs secret more ANGPTL2 compared to NFs (**Figure 8D**). Then we co-cultured CRC cells with NFs or CAFs (**Figure 8E**). Transwell assays showed that compared to NFs, CAFs significantly promotes the metastasis of CRC cells (**Figure 8F**). Subsequently, we overexpressed ANGPTL2 in CAFs by transfecting rANGPTL2 (**Figure 8G**) and co-cultured CAFs with CRC cells. Transwell assays showed that ANGPTL2 overexpression significantly promotes the metastasis of CRC cells (**Figure 8H**). Moreover, the immunoblotting assay exhibited that overexpressing rANGPTL2 in CAFs enhanced the EMT of CRC cells (**Figure 8I**).

### SPP1+macrophages enhance the invasion and metastasis of CRC cells

**Figure 9:**
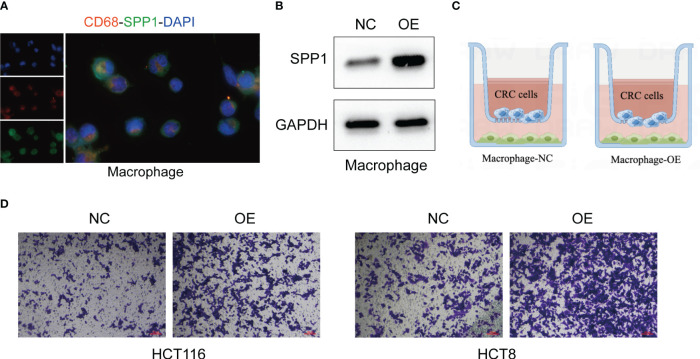
SPP1+macrophages enhance the invasion and metastasis of CRC cells. **(A)** IF assays of the macrophage-specific markers CD68 and SPP1 in macrophages. **(B)** The effect of SPP1 overexpression in macrophages. **(C)** The illustration of co-culturing SPP1-overexpressed macrophages with CRC cells. **(D)** The invasion ability of CRC cells revealed by Transwell assays.

Although the oncogenic role of SPP1^+^macrophages has been reported (27), whether SPP1^+^macrophages regulate the metastasis of CRC cells remains elusive. Then we validated the metastasis-promoting role of SPP1^+^macrophages in CRC based on *in vitro* experiments. We first tested the expression of SPP1 in macrophages (**Figure 9A**). After overexpressing SPP1 in macrophages (**Figure 9B**), we co-cultured them with CRC cells (**Figure 9C**). Transwell assay showed that SPP1 overexpression in macrophages significantly improves the invasion and metastasis abilities of CRC cells (**Figure 9D**).

## Discussion

Liver metastasis is a leading cause of cancer-related deaths in CRC patients (1). Currently, more and more LM-associated regulators have been revealed (28). In the present study, we first selected hundreds of DEGs in LM-CRC compared to primary CRC and found these DEGs are significantly associated with the regulation of endopeptidase activity, blood coagulation, and metabolic processes. Consistently, these DEGs mainly participate in the complement and coagulation cascades and metabolic pathways. The matrix metalloproteinases (MMPs) are common zinc-dependent endopeptidases whose enzymatic activity is cleaving components of the extracellular matrix (ECM), which facilitate tumor cell invasion and metastasis by several mechanisms (29). For instance, MMPs remove physical barriers to invasion through the degradation of ECM macromolecules, such as collagens, laminins, and proteoglycans (30). The association between blood coagulation and cancer metastasis is well recognized. Thrombin, a pleiotropic enzyme, has been found to contribute to cancer metastasis by increasing the adhesive ability of cancer cells (31). Metabolic reprogramming is a hallmark of cancer metastasis in which cancer cells manipulate their metabolic profile to meet the dynamic energetic requirements of the tumor microenvironment (32). For example, cancer cells can preferentially utilize glycolysis or oxidative phosphorylation based on heterogeneous intrinsic or extrinsic factors (32). Cancer metastasis is also regulated by microenvironmental and systemic processes, such as immunosurveillance. Natural killer (NK) cells play an important role in immune responses against cancer metastasis (33). Hassan et al. once reported that NK cells directly interact with circulating tumor cells to control cancer metastasis (34).

Our study identified SPP1, CAV1, ANGPTL2, and COLEC11 as the characteristic DEGs of LM-CRC. SPP1 and ANGPTL2 are also significantly associated with the clinical outcomes of CRC patients. SPP1 and CAV1 have been previously identified as the metastatic markers of LM-CRC through weighted gene correlation network analysis (35). Besides, Xu et al. reported that SPP1 can promote CRC metastasis by activating epithelial-mesenchymal transition (36). Conversely, CAV1 serves as a tumor suppressor in CRC. CAV1 attenuates the migration and invasion of CRC cells by inhibiting the phosphorylation of EGFR (37). COLEC11 is a member of the collectin family of C-type lectins and plays a role in innate immunity through its ability to bind non-self-sugars and to activate the complement through the recruitment of MAPS1 (38, 39). ANGTPL2 is a widely accepted metastasis promoter in various cancers, including CRC (40–43). Given the secreted glycoprotein role of ANGPTL2 protein, Motoyoshi and colleagues uncovered that the serum ANGPTL2 levels are significantly upregulated in breast cancer patients with metastasis and are potential predictors of metastatic breast cancer (26).

Immunohistochemistry analysis revealed that ANGPTL2 and SPP1 proteins are distributed in the TME of CRC tissues. ScRNA-seq analysis demonstrated that ANGPTL2 and SPP1 are markedly enriched in CAFs and macrophages in CRC tissues, respectively. Zhang et al. once found that SPP1 can mediate the M2 polarization of macrophages and upregulate the expression of PD-L1 which attenuates CD4^+^ T-cell activation (44). Recently, a scRNA-seq and spatial analysis discovered that SPP1^+^macrophages are a subgroup of macrophages and interact closely with FAP^+^CAFs in CRC (27). In addition, CRC patients with high FAP or SPP1 expression achieved less therapeutic benefit from an anti-PD-L1 therapy (27). Consistently, our CCI analysis also revealed an interaction between SPP1^+^macrophages and CAFs. M2 macrophages are significantly associated with the metastasis of cancer cells. For example, several tumor-derived exosomal miRNAs can be transferred to macrophages and induced M2 polarization of macrophages which in turn promotes CRC metastasis by enhancing epithelial-mesenchymal transition and secreting VEGF (45). Therefore, it is no wonder that SPP1^+^macrophages are metastasis promoters of LM-CRC. CAFs also play a critical role in cancer metastasis by contributing to ECM deposition and remodeling, extensive crosstalk with cancer cells, and epithelial-to-mesenchymal transition (46). Many CAF-derived factors have been proven to enhance the metastatic ability of cancer cells, such as IL-33 (47), TGF-beta (48), and miR-500a-5p (49). Li et al. previously determined that there are two different CAF subtypes in the CRC tumor microenvironment through scRNA-seq analysis (14). However, with the development of scRNA-seq technology and the increasing sample sizes, more and more CAF subtypes were discovered (50). In this study, we were the first to identify ANGPTL2^+^CAF as a novel CAF subtype and verify that ANGPTL2^+^CAFs release intracellular ANGPTL2 proteins to accelerate the metastasis of CRC cells. Furthermore, we uncovered the specifically enriched gene sets in ANGPTL2^+^CAFs and SPP1^+^macrophages in CRC, such as the HALLMARK_EPITHELIAL_MESENCHYMAL_TRANSITION in ANGPTL2^+^CAFs and the HALLMARK_COMPLEMENT in SPP1^+^macrophages. Epithelial-mesenchymal transition is a process in which epithelial cells acquire mesenchymal features. In cancer, the epithelial-mesenchymal transition is associated with tumor invasion and metastasis closely (51). The complement system that encompasses more than 50 soluble and membrane-bound proteins is a pillar of the innate immune response. Emerging evidence has underscored its relevance in tumor metastasis (52). Our analysis results also revealed that ANGPTL2 and SPP1 levels are positively correlated with the expression of genes in the HALLMARK_EPITHELIAL_MESENCHYMAL_TRANSITION and the HALLMARK_COMPLEMENT in CRC tissues. Here, we validated that ANGPTL2^+^CAFs promote the epithelial-mesenchymal transition of CRC cells. Although we verified SPP1^+^macrophages enhance metastasis of CRC cells based on *in vitro* experiments, whether SPP1^+^macrophages regulate the complement cascades in CRC needs to be further investigated.

In summary, our study selected four characteristic DEGs of LM-CRC and identified ANGPTL2^+^CAFs and SPP1^+^macrophages subtypes as metastasis accelerators of CRC which provided novel potential therapeutic targets for LM-CRC.

## Data availability statement

The datasets presented in this study can be found in online repositories. The names of the repository/repositories and accession number(s) can be found within the article/Supplementary Materials.

## Ethics statement

The studies involving humans were approved by the Ethics Committee of Nanjing First Hospital. The studies were conducted in accordance with the local legislation and institutional requirements. The participants provided their written informed consent to participate in this study.

## Author contributions

XL, SW, and YP: Conceptualization, supervision, funding acquisition, and writing—original draft preparation. XL, JQ, JN, and RG: methodology, software, validation, formal analysis, investigation, data curation. SH and HS: validation and editing. All authors contributed to the article and approved the submitted version.
